# Climate Change and Health: Challenges to the Local Government Environmental Health Workforce in South Australia

**DOI:** 10.3390/ijerph20146384

**Published:** 2023-07-18

**Authors:** Harriet Whiley, James C. Smith, Nicole Moore, Rebecca Burton, Nadia Conci, Helen Psarras, Kirstin E. Ross

**Affiliations:** 1 College of Science and Engineering, Flinders University, Bedford Park, SA 5042, Australia; jim@jamescsmith.com.au (J.C.S.); kirstin.ross@flinders.edu.au (K.E.R.); 2 ARC Training Centre for Biofilm Research and Innovation, Flinders University, Bedford Park, SA 5042, Australia; 3 City of Onkaparinga, Noarlunga Centre, SA 5168, Australia; nicole.moore@onkaparinga.sa.gov.au; 4 Renmark Paringa Council 61 Eighteenth Street, Renmark, SA 5341, Australia; 5 Eastern Health Authority, 101 Payneham Rd, St. Peters, SA 5069, Australia; nconci@eha.sa.gov.au; 6 South Australian Department for Health and Wellbeing, 11 Hindmarsh Sq, Adelaide, SA 5000, Australia

**Keywords:** preparedness, public health, COVID-19, environmental health officer, environmental health practitioner

## Abstract

Climate change is the most urgent and significant public health risk facing the globe. In Australia, it has been identified that Environmental Health Officers/Practitioners (EHOs/EHPs, hereafter EHOs) are a currently underutilized source of knowledge and skills that can contribute to climate change adaptation planning at the local government level. The ability of local government EHOs to utilize their local knowledge and skills in human health risk assessment during a public health emergency was demonstrated through their role in the response to COVID-19. This study used a survey and follow up interviews to examine the roles and responsibilities of EHOs during the COVID-19 pandemic and used the results to examine the potential of the workforce to tackle climate change and health related issues. What worked well, what regulatory tools were helpful, how interagency collaboration worked and what barriers or hindering factors existed were also explored. A workforce review of EHOs in South Australia was also undertaken to identify current and future challenges facing EHOs and their capacity to assist in climate change preparedness. The findings demonstrated that the workforce was used in the response to COVID-19 for varying roles by councils, including in education and communication (both internally and externally) as well as monitoring and reporting compliance with directions. Notably, half the workforce believed they could have been better utilized, and the other half thought they were well utilized. The South Australian Local Government Functional Support Group (LGFSG) was praised by the workforce for a successful approach in coordinating multiagency responses and communicating directions in a timely fashion. These lessons learnt from the COVID-19 pandemic should be incorporated into climate change adaptation planning. To ensure consistent messaging and a consolidated information repository, a centralized group should be used to coordinate local government climate change adaptation plans in relation to environmental health and be included in all future emergency management response plans. The surveyed EHOs identified environmental health issues associated with climate change as the most significant future challenge; however, concerningly, participants believe that a lack of adequate resourcing, leading to workforce shortages, increasing workloads and a lack of support, is negatively impacting the workforce’s preparedness to deal with these emerging issues. It was suggested that the misperception of environmental health and a failure to recognize its value has resulted in a unique dilemma where EHOs and their councils find themselves caught between managing current workload demands and issues, and endeavouring to prepare, as a priority, for emerging environmental health issues associated with climate change and insufficient resources.

## 1. Introduction

Currently, climate change is one of the most significant global public health threats [1]. It has many direct and indirect consequences that impact human health. These include access to clean air, secure shelter and safe drinking water and food. Climate change also impacts the spread of climate sensitive infectious diseases, such as vector borne, foodborne and waterborne diseases [1,2,3,4]. The impact of these diseases is further compounded by population displacement, resulting in reduced access to health services [1] as was seen in the current cholera outbreak affecting over 30 countries, many of which had been free from the disease for decades prior to this current outbreak [5]. It is thought that an increase in the number of people living in extreme poverty without basic access to water and sanitation hardware is the primary cause of the current outbreak. However, it is acknowledged that climate change has also played a critical role, with more frequent and intense weather events such as hurricanes and floods damaging critical water and sanitation infrastructure [5,6]. The outbreak was also exacerbated by increasing temperatures; for example, in Zanzibar, a 1 °C rise in temperature was found to be associated with a 2-fold increase in cholera [7]. Climate change was also suggested to play a role in the emergence and transmission of the novel coronavirus causing Severe Acute Respiratory Syndrome SARS-CoV-2 (COVID-19), with pandemics generally predicted to be more frequent and more severe in the future unless climate changes are mitigated [8].

In Australia, the Federal Department of Health subscribes to the World Health Organization definition of environmental health: “Environmental health addresses all the physical, chemical and biological factors external to a person, and all the related factors impacting behaviors. It encompasses the assessment and control of those environmental factors that can potentially affect health. It is targeted towards preventing disease and creating health-supportive environment” [9,10,11]. This definition acknowledges that environmental health covers a range of complex and multidisciplinary issues and represents a broader, more holistic, approach and understanding. It also moves away from the traditional perspective that environmental health practice is primarily focused on regulation and enforcement. Environmental Health Officers/Practitioners (EHOs/EHPs, hereafter EHOs) hold an appropriate environmental health qualification as determined by the relevant state or territory authority. EHOs work in federal, state and local government, in the defence forces and within private practice; however, the majority are employed by local governments [12]. The focus of local government environmental health officer (EHO) practice is the protection of human health from microbiological, toxicological and physical threats in the natural and built environments [13]. Typically, practice is concerned with the regulation of food safety, accommodation standards, recreational water standards, domestic waste water, waste water recycling and personal care and body art services, and with controlling specific physical environments which have potential for disease spread, such as, mosquito breeding and health nuisances emanating from private domestic or commercial premises [14]. Thus, EHOs with their regulatory powers, location within the community and their skills in human health risk assessment make them the ideal workforce to tackle the health protection aspects of climate change adaptation [8,15,16,17]. In recognition of this, Gerding et al. [18] conducted a review of the current environmental health workforce in the United States to identify their current challenges. They identified key priority areas needed to strengthen the United States environmental health workforce including effective leadership, workforce development, equipment and technology, information systems and data, garnering support through collaboration and developing partnerships. Australia is uniquely vulnerable to extreme climatic and weather events and, as such, climate change adaptation and preparedness is essential to protect our communities [19]. However, a recent study by Smith, Whiley and Ross [16] found that current Australian local government climate adaptation and mitigation planning does not include the human health impacts outside of extreme weather events. This study also found that Australian local government EHOs are an underutilized source of knowledge and skills that can contribute to climate change adaption planning for health protection [16].

The aim of this study was to assess the current South Australian local government environmental health workforce and to identify the current workforce needs and emerging challenges. This study also explored the role of the local government environmental health officers in the response to COVID-19 and identified the lessons learnt regarding useful regulatory tools and mechanisms that supported interagency collaboration as well as any barriers, or hindering factors, that occurred during the response to COVID-19. These lessons can inform both future emergency management aspects of climate change, and climate change adaptation planning for human health impacts. These findings will identify areas of priority for the environmental health workforce to ensure a strong and robust workforce needed for climate change adaptation and preparedness and to protect public health in the future.

## 2. Materials and Methods

This study utilized a mixed method approach and included quantitative and qualitative methods. An online survey was created using Qualtrics^®^ (Supplementary Figure S1). The survey was directed to South Australian Environmental Health Officers (EHOs) and was disseminated initially via a quick response (QR) code that was displayed on screens during a COVID-19 workshop (22 November 2020) run by Environmental Health Australia (the peak environmental health professional organization in South Australia). The survey was then also sent via email on 15 March 2021 to all members on the mailing list of Environmental Health Australia (South Australia) mailing list. A total of 66 EHOs completed the survey and 23 provided their email address indicating a willingness to be contacted for a follow up interview. However, given that participants could skip a question without answering, not all questions received responses from all 66 participants. Sixty nine percent (45/65) of the respondents were generalist field based EHOs, with 23% (15/65) consisting of managers or team leaders and the remaining five responses were from EHO specialists, or individuals in other environmental health roles. Sixty five percent (42/65) were from metropolitan and 31% (20/65) were from regional local government environmental health programs, with three respondents selecting “other”.

An email was sent to the 23 survey participants who indicated that they would be willing to answer further questions through a follow up phone interview. Out of these twenty-three, eight responded indicating a willingness to participate in a follow up interview with no response received from the remaining fifteen individuals. These interviews were conducted over the phone following the script (Supplementary Figure S2) which was developed to elicit broader or deeper responses than those possible in the survey. This study (both the survey and follow up interviews) was approved by the Flinders University Human Social and Behavioural Research Committee, (approval number 2798). Survey data was analysed and graphed using SPSS (Microsoft).

## 3. Results

### 3.1. Survey Results: Participant Demographics and Workplace Profiles

The survey results demonstrated that the survey participants consisted of an even distribution of age demographics with slightly more females (60%, 40/66) compared with males (38%, 25/66), with one person preferring not to say (as shown in Supplementary Figure S3). Participants were found to be highly experienced with 61% indicating they had more than ten years’ experience working in the environmental health profession.

Most of environmental health teams had 1–5 employees (58%), followed by teams with 5–10 employees (40%), and one respondent worked in an environmental health workforce of 11–25 employees. Unsurprisingly, most of the workforces with higher numbers of EHOs were in metropolitan local government environmental health teams with only two respondents indicating they worked for a rural local government environmental health program with 5–10 environmental health employees. It was also found that there was a significant amount of movement between environmental health workplaces, with 43% of respondents indicating they have been with their current employer for less than two years. Over 80% of the respondents were on permanent contracts and 69% worked full time. Fifty-four percent travel for less than 20 min to work and only 12% travel more than an hour. When asked about remuneration, 64% of respondents indicated they thought they were getting paid an amount commensurate with their role and experience, and 36% indicated that they were not. This was irrespective of the number of years’ experience working in environmental health (Supplementary Figure S5).

Most of the respondents reported being satisfied with their current employer, with 63% indicating that they have a career with their current employers and will stay there for the foreseeable future. Seventeen percent indicated that they were looking to leave the environmental health profession within the next two years. Thirty percent of respondents expected to keep working in environmental health for more than ten years and 11% indicated that that would be working in environmental health for the next 0–2 years. When this was sorted according to number of years’ experience (Supplementary Figure S6) and where the respondent worked (e.g., metropolitan or rural) no trends were observed (Supplementary Figure S7), with the exception that 100% of EHOs new to the workforce (1–2 years’ experience) indicating they planned to stay for 10 plus years.

Respondents were asked how important a series of factors were at keeping them at their current organization. When just considering the number of ‘very important’ responses, work/life balance was identified as the most important factor keeping an individual at an organization with 60% identifying this as ‘very important’. When combining ‘very important’ and ‘important’ responses over 90% of respondents selected work–life balance, “I like the work I do”, “pay” and “relationships at work”; 89% selected “belief that my work is valuable”; 80% “employment security”; 60% “[working] close to home”; and 58% selected “employment conditions”.

### 3.2. Survey Results: Workforce Challenges

In terms of activities undertaken by EHOs, thirty percent of respondents indicated that they performed activities that were outside the specific scope of environmental health. No trend was observed with these responses when they were sorted according to metropolitan or rural local government environmental health teams (Supplementary Figure S4). In the free text box, a range of activities beyond environmental health were identified as being currently undertaken by the environmental health workforce. These included roles in community safety and customer service (1 respondent), environment and sustainability (3), administration and contract management (4), immunization (1), waste management (2), supported residential facilities (SRFs) (1) and nuisance compliance (5).

When asked specifically about current workforce challenges, participants referred to three areas: support for conducting their roles and responsibilities, financial resources and the perceived value of environmental health within their organizations.

Regarding the level of support received to perform their roles and responsibilities, 32% of respondents indicated that they did not have enough support. Proportionately, metropolitan local government environmental health teams felt more supported compared with rural teams. When asked to identify what additional support they needed, the most common answers provided in the free text box were around more EHO staff members/more FTE [full-time equivalent] positions/realistic workload (n = 6), more administrative support (n = 7) and more support/better understanding of environmental health and its role from management (n = 5). Other responses included emotional support (n = 1) and more training/professional development (n = 2).

Financial resource issues were seen to be one of the biggest barriers to ensuring an adequate workforce and related to a lack of income generation: “A lack of income through environmental health as a general thing. It’s hard to promote to council the need for additional resources probably when we can’t offset that by other income streams in the work that we are doing”.

The majority of respondents answered that they believed that environmental health was ‘somewhat’ valued (33%) in their organization. Interestingly, of the other respondents, half felt valued to a very high degree (13%) and the other half to a very low degree (8%). When these data were sorted based on the type of workplace, no difference was observed in the answers provided by metropolitan compared with rural local government environmental health teams.

### 3.3. Interview Results: Workforce Challenge

In the follow up interviews, participants were asked “Now thinking beyond COVID-19–more broadly what do you see as the biggest challenges facing the environmental health workforce in the future?” (Supplementary Figure S2). Two participants indicated that inadequate resourcing and budget constraints was the greatest issue, with one of these respondents discussing how this led to workload issues and the other suggesting budget constraints were due to a lack of recognition of the importance of environmental health from within the organization. One participant suggested that the budget constraints and resources issues were due to the lack of income generation by the environmental health team. This participant also suggested that if the South Australian Health Department (SA Health) required environmental health to be involved in a broader range of local governments activities (planning, development, etc.) and required them to report on these issues, then the councils would be forced to provide adequate resources: “So whatever SA Health requires for us to report, or councils to report on, councils will put that at the top of their agenda in terms of EHO staff.” However, currently this is not the case and reporting is often streamlined to simply report numbers of inspections and other compliance activities.

Three participants indicated that attraction and retention of a suitably qualified workforce was the biggest challenge. Another participant indicated that the under recognition of the profession from both within and outside of council was the greatest challenge facing the workforce in the future. This participant commented that the misperception of environmental health by both the community and within the organization had a substantial impact on resources. This was attributed to a number of factors, including the lack of income generation by environmental health teams, the broadness of scope of the environmental health role, fragmentation of tasks and workforce attraction and retention issues.

The final two participants answered that climate change (and emerging public health issues relating to climate change) was the biggest challenge. When questioned further, the participants expanded on this answer and identified that part of this challenge was linked to resourcing and the need for an increased resilient and skilled environmental health workforce to address the emerging environmental health issues arising from climate change as well as increased population growth and urban development.

### 3.4. Survey Results: Workforce Response to COVID-19

In the free text box provided, respondents indicated that they had been involved in a number of activities relating to their organization’s response to COVID-19. The most common responses were education and communication (internally and externally) (n = 23), and monitoring and reporting compliance with directions (n = 19). Other responses included completing internal risk assessments, internal planning and response, supporting airport security procedures and border closures.

However, 53% of respondents indicated that they felt they could have been better utilized by their organization with no difference observed between rural and metropolitan local government respondents. For those respondents who answered ‘yes’, a free text box was provided for them to provide some examples of the activities they could have undertaken. This included contact tracing (n = 6), greater involvement/leadership in internal decision making and response (n = 13) and more engagement with aged care facilities (n = 1) and the community through education (n = 1).

The response to the COVID-19 pandemic has highlighted that the environmental health profession possesses skills and knowledge that are transferable to other areas in times of need. This was shown by 86% of respondents agreeing, or somewhat agreeing, that during COVID-19 they were tasked with responsibilities outside their normal scope of work. Notwithstanding, 97% (agreed or somewhat agreed) acknowledged that these additional responsibilities had an environmental health focus. Eighty one percent of the respondents felt they were adequately trained to complete these tasks (agreed or somewhat agreed).

Overall, workplace safety during COVID-19 was taken seriously by employers, with 70% of respondents (agreed or somewhat agreed) indicating that they were regularly asked about safety concerns and 76% (agreed or somewhat agreed) stated that they received frequent communications about workplace safety. Encouragingly, 89% (agreed or somewhat agreed) were provided with adequate personal protection equipment (PPE) by their organization. Sixty seven percent indicated they had a sufficient number of employees to conduct the work needed (agreed of somewhat agreed) and 86% (agreed or somewhat agreed) stated they had access to adequate environmental health COVID-19 situations reports and updates.

When asked about questions relating to burnout, emotional exhaustion, frustration, feeling worn out or tired and having enough time for leisure, respondents identified that overall, burnout symptoms increased slightly during COVID-19 (with 23% of respondents answering to a high or very high degree in answer to the question “Did you feel burnt out because of work?” before COVID-19, increasing to 26% during COVID-19), as did emotional exhaustion (with 20% of respondents answering to a high or very high degree in answer to the question “Was your work emotionally exhausting?” before COVID-19, increasing to 31% during COVID-19). This differed from respondents’ answers to questions related to frustration and being worn out and tired, which decreased during COVID-19. Interestingly, feeling worn out at the end of the day decreased during COVID-19 by 11% (from 44% to 33% of respondents answering “often” or “always” to the question “Did you feel worn out at the end of the day?), as did feeling frustrated (a decrease from 23% to 21% of respondents answering “often” or “always” to the question “Did your work frustrate you?). Nine percent of respondents indicated that they rarely had enough energy for leisure (increased to 15% during COVID-19), and none indicated that they never have enough energy.

### 3.5. Interview Results: Workforce Response to COVID-19

In the follow up interviews all eight participants indicated that they were involved in the response to COVID-19. The specific activities identified during the interviews included education, monitoring, reporting and inspections to ensure compliance with directions around social distancing and closures of nonessential businesses. Two participants also identified that they supported food businesses in the transition to take away meals and infection control strategies and one participant was involved in border closures.

During these interviews, six participants identified that LG EHOs obtained advice primarily from the Local Government Functional Support Group (LGFSG). The support offered by the LGFSG was almost universally praised and included inviting EHOs to attend regular LGFSG meetings to seek clarification or ask questions. The LGFSG would also provide regular emails and facts sheets to clarify certain aspects of the legislation. The LGFSG would seek further clarification from SA Health and the South Australian Police (SAPOL) and then provided the response back to the EHOs. This coordinated approach to communication was found to be significantly more helpful than EHOs going straight to SA Health or SAPOL which could result in receiving conflicting directives or responses not delivered in a timely way. The speed at which directives changed was a big challenge and due to this the daily LGFSG meetings were seen as valuable: “The LGA meetings every morning was quite good just to update what was happening and what they wanted us to do”.

## 4. Discussion

### 4.1. Workforce Demographics

The results from this study showed that there has been a significant shift in the demographic distribution to a more feminized and older workforce, since the last review of the South Australian Environmental Health workforce, which was conducted in 2010 [20]. This gender shift trend from male to female was also observed in the younger cohort of the Western Australian environmental health workforce, with a recent survey showing that 60% of the under 40 year olds were female [21]. However, in both this South Australian study and in the Western Australian study, the older workforce cohort (over 50 year old) was still male dominated, with only 33% and 24% women, respectively [21].

The profession is also more experienced, with 61% compared with 48% in 2010 indicating they had more than 10 years’ experience. The majority of respondents hold university level qualifications (82%) with 18% (12/66) indicating they were TAFE qualified. This shift in education levels was also shown in the recent survey in Western Australia with 94.9% of participants indicating that they held undergraduate or post graduate qualifications in environmental health [21]. This evidence demonstrating that the profession is highly educated supports the argument that they have the necessary skills and expertise needed to drive local government climate change adaptation and mitigation planning. The consistent approach to education is also a strength of the Australian Environmental Health Workforce in comparison to the workforce in the US. [18] argued that one of the factors hindering the environmental health workforce in the United States was the varied educational and credentialing requirements. This led to a poorly defined professional identity that ultimately has a detrimental impact on the recognition and awareness of the profession. In comparison, all Australian environmental health qualifications are accredited by Environmental Health Australia (the peak professional body) using the Environmental Health Officer Skills and Knowledge Matrix developed by the Environmental Health Standing Committee (enHealth) [13]. Currently, there are a range of both undergraduate and postgraduate degrees that meet the accreditation policy [22]. However, there is still room for improvement as the need to hold an accredited environmental health degree to be an authorized environmental health officer under the relevant State/Territory Public Health Acts varies between the different State/Territory governments. It is also interesting to note that despite Australia’s standardized education approach compared with the United States, the issue of a poorly defined professional identity is still apparent with varying survey responses to what was perceived to be in the scope of practice.

### 4.2. Future Workforce Challenges

There seemed to be a common view, from both this study and the USA [18], that there is a need to develop a resilient and skilled environmental health workforce to meet both current environmental health challenges posed by population growth and development, and the future challenges posed by climate change. However, there appears to be two hindrances to developing this future workforce. The first of these comes from perceptions of what is seen to be the scope of local level environmental health practice and activities by the practitioners themselves and their managers. Historically, EHOs have perceived their roles as a narrowly regulatory one focused exclusively on statutory enforcement activities, although the 1988 WHO Second International Conference on Health Promotion in Adelaide did open opportunities to expand practice into health promotion and public health planning [23]. With the introduction of the New Environmental Health in 1999 it was noted that there continued to be a strong focus on enforcement and monitoring of legislative requirements by practitioners and a study in 2021 indicated that this focus was still in evidence [24]. Seemingly local environmental health officers define their professional role by the legislative obligations imposed on councils rather than on the competencies required to conduct this practice [25] indicating the lack of a self-determined definition of practice. If this is the case it would explain why some EHOs thought that certain activities, that is, those lacking a statutory obligation like community safety and customer service, environment and sustainability, administration and contract management were outside the scope of environmental health; however, there is a lack of consistency as immunization, waste management, supported residential facilities and nuisance compliance were also seen to be outside the scope of environmental health but these activities do have statutory obligations on local councils. What are consistent with the statutory orientation of practitioners are suggestions that SA Health mandate compliance requirements for those broader activities, like planning, thus providing a statutory obligation and it was perceived that this would then provide a driver for more resources.

### 4.3. Lessons Learnt from COVID-19

The SA EHOs surveyed and interviewed in this study reported participation in a range of activities related to the COVID-19 pandemic response. This demonstrates a willingness to undertake major activities outside their normal scope of work. Furthermore, these activities were not based on statutory obligations and included community education and communication, completion of risk assessments and internal planning. With 53% of participants indicating that they felt they could have been better utilized in contact tracing, leadership in internal decision making and response, more engagement with aged care facilities and the community through education amongst others. It appears that the pandemic demonstrated to EHOs the need for a range of skills and activities not necessarily based in statute, in addition to the statutory activities, all of which were required to meet the public and environmental health challenges at the community level.

**Figure 1:**
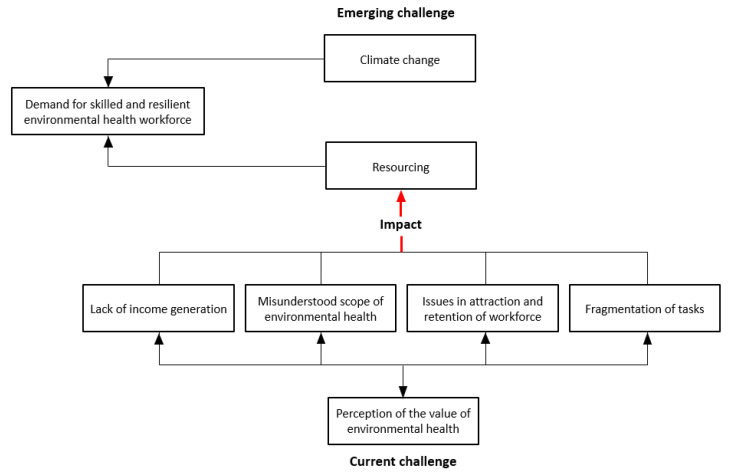
The South Australian local government environmental health challenges as identified from survey and interview responses.

A success of the COVID-19 response in South Australia was the multiagency collaboration as coordinated by the LGFSG. This group was praised by the LG EHO for coordinating information to ensure consistent messaging. However, the LGFSG had a defined and narrow scope of practice, and this multiagency collaboration and communication did not necessary translate beyond the specific activities considered within the scope of this group. In the United States, Ryan, et al. [26] highlighted the need for interdisciplinary public health solutions to respond and recover successfully from coronavirus disease 2019 (COVID-19). However, one of the most significant impediments to this was the lack of awareness of the environmental health workforce connections and capabilities. This is also seen in Australia with the second hindrance to developing a resilient and skilled workforce relating to the perceived value placed on environmental health by councils. The majority (33%) of surveyed EHOs perceived that their employer organizations only somewhat value environmental health with a split in the other respondents varying from valued to ‘a very high degree’ and valued to ‘a very low degree’. There seemed to be a suggestion that this value, in part, was related to a lack of income generation by environmental health activities with an implication that unless service generated income it was not as valued as a service that did so. The lack of value may also reflect a poor understanding of environmental health by council management [24] and a failure to realize the potential of EHOs to have a role in future challenges [16] which may be reinforced by a lack of a common perception of environmental health by practitioners and a failure to advocate for the science. This in turn, would have impacts on recruitment and retention of practitioners. These factors are interrelated with the increasing scope of work preventing EHOs from participating in the leadership and collaborative partnership activities needed to raise the profile. This in turn hinders the acquisition of additional resources needed to manage workload issues arising through the increased scope of practice, which in turn impacts on workforce retention and the ability of the workforce to contribute to further climate change adaptation planning. The interconnectedness of South Australian local government environmental health workforce challenges and how they related to climate change is captured in Figure 1 below.

## 5. Conclusions

Local Government Environmental Health Officers (EHOs) in Australia, and elsewhere, have been identified as the ideal workforce to tackle many of the health protection aspects of climate change adaptation. As such, there is a need to ensure a resilient and skilled environmental health workforce to meet current needs and future challenges. However, in South Australia the current workforce is facing a lack of adequate resources leading to workforce shortages and increasing workloads. This appears to be a result of two hindering factors. The first relates to the perceptions of what is seen to be the scope of local government environmental health practice. The second relates to a lack of recognition and value of environmental health by their councils. These factors are interrelated with high workloads preventing EHOs from participating in the leadership and collaborative partnership activities needed to raise their profile, which in turns hinders the acquisition of additional resources leading to workforce retention issues.

## Data Availability

Selected survey data is provided in supplementary materials. The full data set and interview transcripts are unavailable due to ethical restrictions.

## Supplementary Materials

The following supporting information can be downloaded at: https://www.mdpi.com/article/10.3390/ijerph20146384/s1, Figure S1: Survey question; Figure S2: Follow up interview questions; Figure S3: Age and gender of participants; Figure S4: “Do you perform roles other than environmental health as part of your current job?” compared with where respondent works (n = 64) (* no response from state government or private company); Figure S5: “Do you think you are getting paid an amount commensurate with your role and experience?” compared with number of years of working in environmental health (n = 64); Figure S6: “How many years do you expect to continue working in an environmental health role?” compared with number of years of experience (n = 66); Figure S7: “How many years do you expect to continue working in an environmental health role?” compared with where respondent works (* no response from state government or private company).
