# Supplementary material for: Climate Change and Health: Challenges to the Local Government Environmental Health Workforce in South Australia
Source: Int J Environ Res Public Health. 2023 Jul 18;20(14):6384. doi: 10.3390/ijerph20146384 (PMC10379352; doi:10.3390/ijerph20146384)
Supplement: Supplementary file 1 [file ijerph-20-06384-s001.zip › supplementary files/Supplementary F1.pdf]

Flinders UNIVERSITY

Information sheet eha 2020 Information sheet - click here if you would like to download information about this survey

*This research project has been approved by the Flinders University Social and Behavioural Research Ethics Committee (Project number: 2798).*
*For more information regarding ethical approval of the project only, the Executive Officer of the Committee can be contacted by telephone on (08) 8201 3116, by fax on (08) 8201 2035, or by email to*
*human.researchethics@flinders.edu.au*

**Demographics**

Which age group are you represented by?

- 20 - 30 years
- 31 - 40 years
- 41 - 50 years
- 50+ years

How would you describe your gender?

- Male
- Female
- Prefer to self describe as ______
- Prefer not to say

How many years have you been working in environmental health?

- 0 - 1 years
- 1 - 3 years
- 3 - 5 years
- 5 - 10 years

- 10+ years

What environmental health qualifications do you hold?

- Pre-degree (TAFE)
- A bachelor's degree
- A postgraduate degree
- Other (please specify) ______

From what institution did you complete your environmental health qualification?

- Flinders University
- Western Sydney University
- Central Queensland University
- Griffith University
- University of the Sunshine Coast
- Edith Cowan University
- Queensland University of Technology
- Curtin University
- Swinburne University of Technology
- TAFE
- Overseas
- Other (please specify) ______

What year did you graduate?

______

**Recruitment, mobility and retention**

Where do you work?

- New South Wales

- Queensland
- South Australia
- Tasmania
- Victoria
- Western Australia
- Australian Capital Territory
- Northern Territory
- Outside Australia (please specify)
- Other (please specify)

What is your current role?

- Manager
- Coordinator/Team Leader
- EHO general
- EHO specialist (please specify)
- Other (please specify)
- No longer work in environmental health (please specify why not)

Where do you work?

- Metropolitan local government environmental health program
- Regional local government environmental health program
- State government environmental health program
- Private company
- Other (please specify)

How large is the environmental health workforce in your workplace?

- 1-5
- 5-10

- 11-25
- 25+

How long have you worked for your current employer?

- 0-2 years
- 3-5 years
- 6-10 years
- 10+ years

What is the nature of your employment contract?

- Permanent
- Casual
- Contract (term 0 to 1 year)
- Contract (term 0 to 3 years)
- Contract (term over 3 years)
- Employed by contractor

What hours do you work?

- full time
- 30-34 hours per week
- 16-29 hours per week
- Less than 16 hours per week

What is your travel time to work?

- 0-20 mins
- 20-40 mins
- 40 mins - 1 hr
- 1hr+

Do you think you are getting paid an amount commensurate with your role and experiences?

- Definitely yes
- Probably yes
- Probably not
- Definitely not

How important are each of these factors in keeping you at your current organisation?

| | very unimportant | unimportant | neither | important | very important |
|---|---|---|---|---|---|
| 'I like the work I do' | ○ | ○ | ○ | ○ | ○ |
| Employment conditions | ○ | ○ | ○ | ○ | ○ |
| Work / life balance | ○ | ○ | ○ | ○ | ○ |
| Relationships at work | ○ | ○ | ○ | ○ | ○ |
| Employment Security | ○ | ○ | ○ | ○ | ○ |
| Pay | ○ | ○ | ○ | ○ | ○ |
| It is close to home | ○ | ○ | ○ | ○ | ○ |
| My belief that the work is valuable for public health protection | ○ | ○ | ○ | ○ | ○ |

Please respond to questions regarding the **intensity** of work-related burnout symptoms (please answer how you felt **before** COVID-19)

| | To a very low degree | To a low degree | Somewhat | To a high degree | To a very high degree |
|---|---|---|---|---|---|
| Was your work emotionally exhausting? | ○ | ○ | ○ | ○ | ○ |
| Did you feel burnt out because of your work? | ○ | ○ | ○ | ○ | ○ |

| | To a very low degree | To a low degree | Somewhat | To a high degree | To a very high degree |
|---|---|---|---|---|---|
| Did your work frustrate you? | ○ | ○ | ○ | ○ | ○ |

Please respond to questions regarding the **frequency** of work-related burnout symptoms (please answer how you felt **before** COVID-19)

| | Never | Rarely | Somewhat | Often | Always |
|---|---|---|---|---|---|
| Did you feel worn out at the end of the working day? | ○ | ○ | ○ | ○ | ○ |
| Were you exhausted in the morning at the thought of another day at work? | ○ | ○ | ○ | ○ | ○ |
| Did you feel that every working hour is tiring for you? | ○ | ○ | ○ | ○ | ○ |
| Did you have enough energy for family and friends during leisure time? | ○ | ○ | ○ | ○ | ○ |

Please select the response which best represents you:

- ○ I have a career here and will stay for the foreseeable future
- ○ I will probably move on within the next year or two but stay in environmental health
- ○ I will probably move on within the next year or two and leave the environmental health profession
- ○ I will be looking at leaving soon but staying in the environmental health profession
- ○ I will be looking at leaving soon and leaving the environmental health profession

How many years do you expect to continue working in an environmental health role?

- ○ 0 - 2 years
- ○ 3 - 5 years
- ○ 6 - 10 years
- ○ 10 + years

- Dont know

Do you perform roles other than environmental health as part of your current job?

- Yes (please specify) ____
- No

Do you have adequate support to carry out your role?

- Yes
- No (please specify what additional support you need) ____

Do you feel environmental health is valued in your organisation?

- To a very low degree
- To a low degree
- Somewhat
- To a high degree
- To a very high degree

Please list any strategies you use to raise the profile of environmental health in your organisation.

____

## Professional development

Have you undertaken professional development/training activities since commencing work?

- Yes (please specify what courses)
- No (please specify why not)

What areas would you like more professional development training in?

How would you like professional development/training activities delivered?

**Environmental health's role in the response to COVID19**

How has the environmental health workforce in your organisation been involved in the COVID-19 response?

Do you feel you could have been better utilized in the response to COVID-19?

○ Yes (please give some examples)

[ ]

○ No

Please indicate your level of agreement with the following statements regarding your organisation's COVID-19 response activities:

| | Disagree | Somewhat disagree | Somewhat agree | Agree | N/A |
| --- | --- | --- | --- | --- | --- |
| Employees are regularly asked about safety concerns | ○ | ○ | ○ | ○ | ○ |
| I have been tasked with responsibilities outside my normal scope of work | ○ | ○ | ○ | ○ | ○ |
| I have been adequately trained to complete my assigned duties | ○ | ○ | ○ | ○ | ○ |
| My responsibilities have an environmental health focus | ○ | ○ | ○ | ○ | ○ |
| My organisation provides me with adequate PPE to use in the field | ○ | ○ | ○ | ○ | ○ |
| There are a sufficient number of employees to conduct the work needed | ○ | ○ | ○ | ○ | ○ |
| I have access to adequate EH COVID-19 situation reports/updates | ○ | ○ | ○ | ○ | ○ |

| | Disagree | Somewhat disagree | Somewhat agree | Agree | N/A |
|---|---|---|---|---|---|
| I have access to adequate EH COVID-19 response technical information/guidance resources | ○ | ○ | ○ | ○ | ○ |
| There are frequent communications about safety in the workplace | ○ | ○ | ○ | ○ | ○ |
| I have been tasked with responsibilities outside my normal scope of work | ○ | ○ | ○ | ○ | ○ |
| I have been adequately trained to complete my assigned duties | ○ | ○ | ○ | ○ | ○ |

Did your feeling of burnout increase **during COVID19**? Please respond to the questions below – this time considering the **intensity** of your work-related burnout symptoms during COVID19.

| | To a very low degree | To a low degree | Somewhat | To a high degree | To a very high degree |
|---|---|---|---|---|---|
| Is your work emotionally exhausting? | ○ | ○ | ○ | ○ | ○ |
| Do you feel burnt out because of your work? | ○ | ○ | ○ | ○ | ○ |
| Does your work frustrate you? | ○ | ○ | ○ | ○ | ○ |

Did your feeling of burnout increase **during COVID19**? Please respond to the questions below – this time considering the **frequency** of your work-related burnout symptoms during COVID19.

| | Never | Rarely | Sometimes | Often | Always |
|---|---|---|---|---|---|

| | Never | Rarely | Sometimes | Often | Always |
|---|---|---|---|---|---|
| Do you feel worn out at the end of the working day? | ○ | ○ | ○ | ○ | ○ |
| Are you exhausted in the morning at the thought of another day at work? | ○ | ○ | ○ | ○ | ○ |
| Do you feel that every working hour is tiring for you? | ○ | ○ | ○ | ○ | ○ |
| Do you have enough energy for family and friends during leisure time? | ○ | ○ | ○ | ○ | ○ |

What areas specifically related to the COVID19 response that you would like additional training or education in?

Would you be happy for us to contact you to discuss your answers in more detail?

○ No

○ Yes (please provide your email address)

Powered by Qualtrics
