# Supplementary figures and images for: Climate Change and Health: Challenges to the Local Government Environmental Health Workforce in South Australia
Source: Int J Environ Res Public Health. 2023 Jul 18;20(14):6384. doi: 10.3390/ijerph20146384 (PMC10379352; doi:10.3390/ijerph20146384)

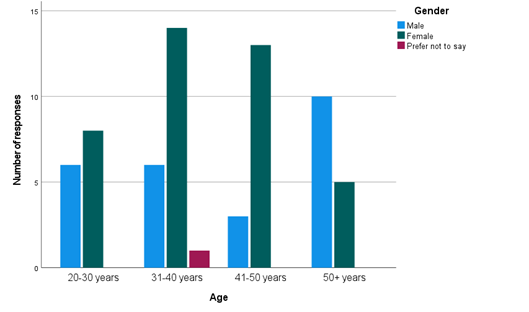

Supplement: Supplementary file 1 [file ijerph-20-06384-s001.zip › supplementary files/S3.png]

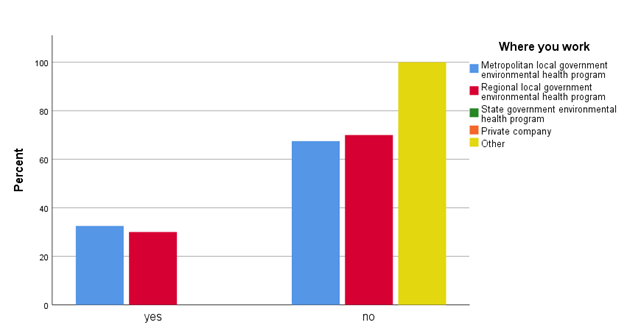

Supplement: Supplementary file 1 [file ijerph-20-06384-s001.zip › supplementary files/S4.png]

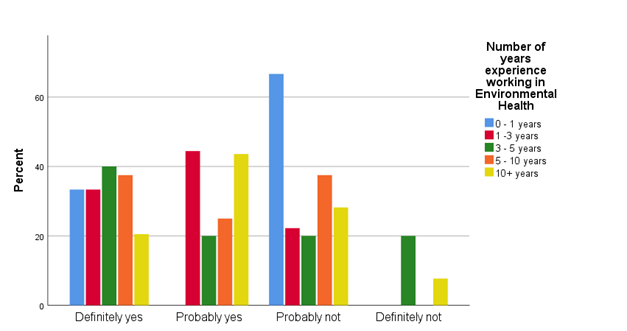

Supplement: Supplementary file 1 [file ijerph-20-06384-s001.zip › supplementary files/S5.png]

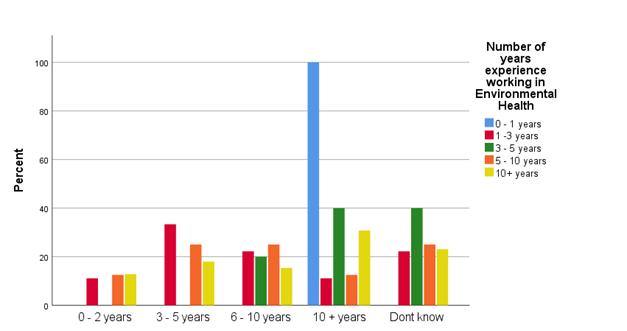

Supplement: Supplementary file 1 [file ijerph-20-06384-s001.zip › supplementary files/S6.png]

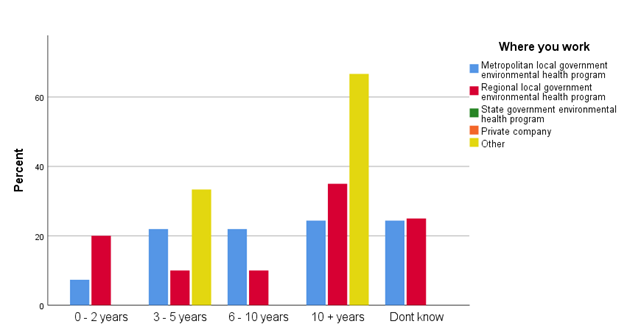

Supplement: Supplementary file 1 [file ijerph-20-06384-s001.zip › supplementary files/S7.png]

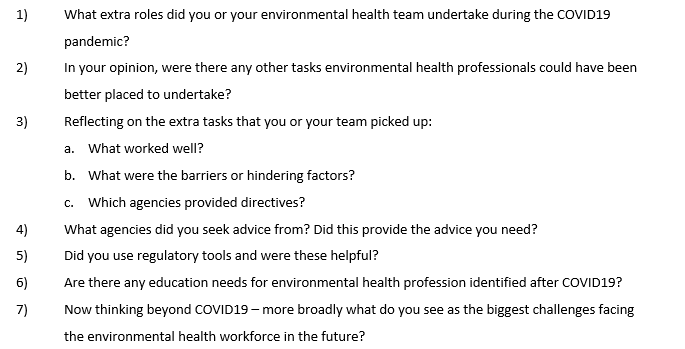

Supplement: Supplementary file 1 [file ijerph-20-06384-s001.zip › supplementary files/Supplementary F2.PNG]
